# Paracorporeal Lung Devices: Thinking Outside the Box

**DOI:** 10.3389/fped.2018.00243

**Published:** 2018-09-05

**Authors:** Timothy M. Maul, Jennifer S. Nelson, Peter D. Wearden

**Affiliations:** ^1^Department of Cardiac Surgery, Nemours Children's Hospital, Orlando, FL, United States; ^2^Department of Biomedical Engineering, University of Pittsburgh, Pittsburgh, PA, United States; ^3^Department of Cardiothoracic Surgery, University of Pittsburgh, Pittsburgh, PA, United States

**Keywords:** paracorporeal, lung assist, VAD, oxygenator, hybrid

## Abstract

Extracorporeal Membrane Oxygenation (ECMO) is a resource intensive, life-preserving support system that has seen ever-expanding clinical indications as technology and collective experience has matured. Clinicians caring for patients who develop pulmonary failure secondary to cardiac failure can find themselves in unique situations where traditional ECMO may not be the ideal clinical solution. Existing paracorporeal ventricular assist device (VAD) technology or unique patient physiologies offer the opportunity for thinking “outside the box.” Hybrid ECMO approaches include splicing oxygenators into paracorporeal VAD systems and alternative cannulation strategies to provide a staged approach to transition a patient from ECMO to a VAD. Alternative technologies include the adaptation of ECMO and extracorporeal CO_2_ removal systems for specific physiologies and pediatric aged patients. This chapter will focus on: (1) hybrid and alternative approaches to extracorporeal support for pulmonary failure, (2) patient selection and, (3) technical considerations of these therapies. By examining the successes and challenges of the relatively select patients treated with these approaches, we hope to spur appropriate research and development to expand the clinical armamentarium of extracorporeal technology.

## Introduction

For the past 40 years, pediatric extracorporeal life support (ECLS) has utilized innovative strategies, novel technology, or repurposed adult medical devices to provide life-saving care to this unique patient population. Prior to the availability of commercial equipment, practitioners made their cannulae, bladder systems, and customized circuits in-house. Commercially available devices for adults have continued to enter the clinical arena, leaving pediatric providers little choice but to use them off-label and at the edge of their intended operational limits. The relative dearth of commercial devices and solutions in pediatrics is a function of the smaller size of the patients and its anatomic challenges, and the smaller sized population and its financial market challenges. Since 2012, the number of adult applications of extracorporeal membrane oxygenation (ECMO) has overtaken the combined use in pediatric and neonatal patients (1). Accommodating the range of patient sizes present in pediatric medicine (from <2 kg premature neonates to 80+ kg teenagers), is a difficult task for medical device designers. Producing one device for a narrow population would further limit the patient population served. However, producing an array of devices can exponentially complicate and increase production and regulatory costs. Because these factors are not expected to change in the near future, clinicians have continued to adapt available technology to meet the needs of their patients and published case reports to share their experience.

Venoarterial (VA) and venovenous (VV) ECMO are gaining acceptance as a standard of care for lung failure refractory to conventional treatment (2, 3), and VADs are increasingly utilized for pediatric heart failure (4). However, there are patients whose anatomy, specific etiology of lung failure, or concomitant requirements for other organ support devices have led practitioners to look for new ways to apply the underlying ECMO and VAD technology to treat their patients. This chapter will explore these unique patient populations, highlight the various approaches that have been used for supporting their lung failure, and provide recommendations and insights for future applications. It should be noted that, like most VAD and ECMO devices applied to pediatrics, these approaches are off-label and should be reserved for the more skilled practitioners of the underlying technologies. Experienced teams are more likely to be aware of the many, sometimes subtle, complications (e.g., infection, stroke, renal insufficiency, etc.) and challenges associated with mechanical circulatory support in children that can lead to poor outcomes. Three general approaches will be discussed: pumpless oxygenators or paracorporeal lung assist (PLA), ventricular assist devices with oxygenators (VAD+Oxy), and adaptation of extracorporeal CO_2_ removal (ECCO_2_R) devices for pediatric ECMO.

## Paracorporeal lung assist

Pediatric patients requiring ECMO as bridge to lung transplantation tend to fare worse than their adult counterparts (5, 6). The reasons for this finding are likely multifactorial. A perceived need for more sedation in children compared to adults on ECMO can lead to lower pre-transplant mobility. A smaller donor pool for pediatric lungs also makes wait times comparatively longer in children than in adults (7). In addition, long-term ECMO may lead to more clinically significant complications including stroke, hemolysis, and infection (8–10). The potential for PLA, essentially an artificial lung, has been the hope as a bridge for lung transplantation or as potential destination therapy for over 30 years (11). The advent of low resistance ECMO oxygenators (<50 mmHg pressure drop across the oxygenator at peak flow), has inspired clinicians to utilize these in a PLA configuration, particularly for young patients presenting for lung transplantation with pulmonary hypertension.

### Oxygenators

**Figure 1:**
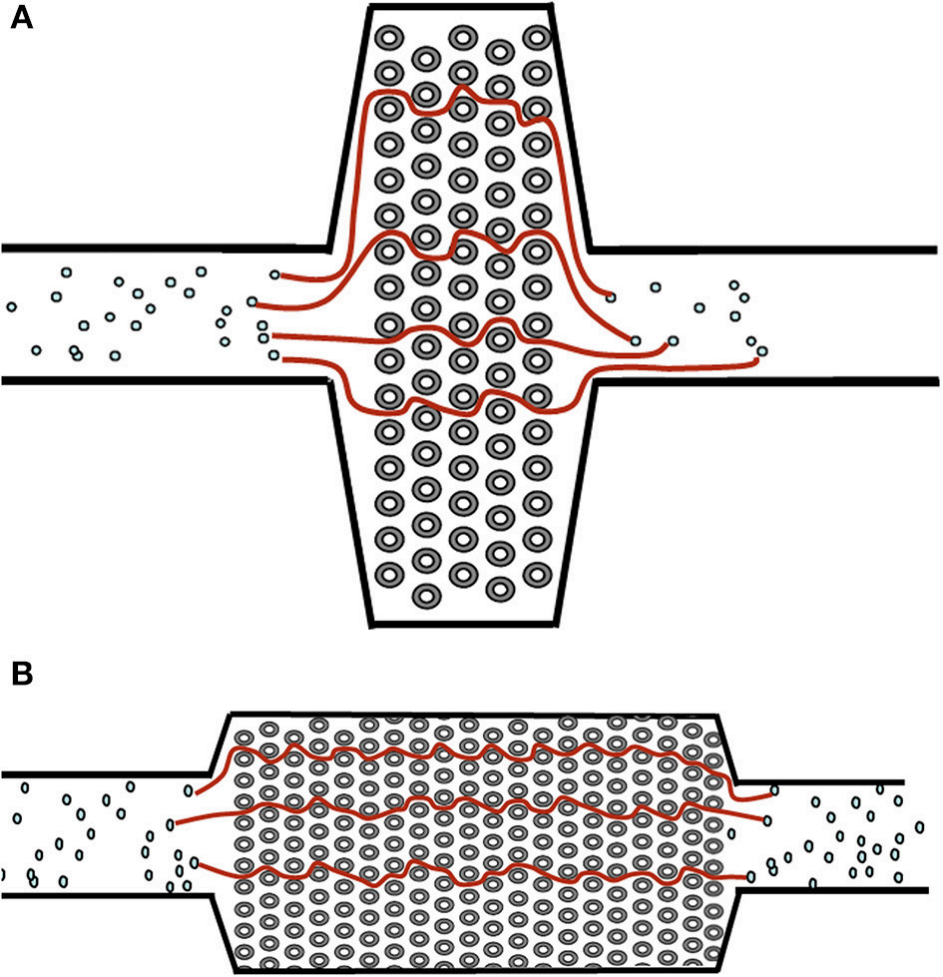
Examples of a short path **(A)** and long path **(B)** oxygenator design. Streamlines denote the potential path of blood cells through the oxygenator. The longer the path line, the higher the pressure drop (and thus higher resistance) of the oxygenator.

**Figure 2:**
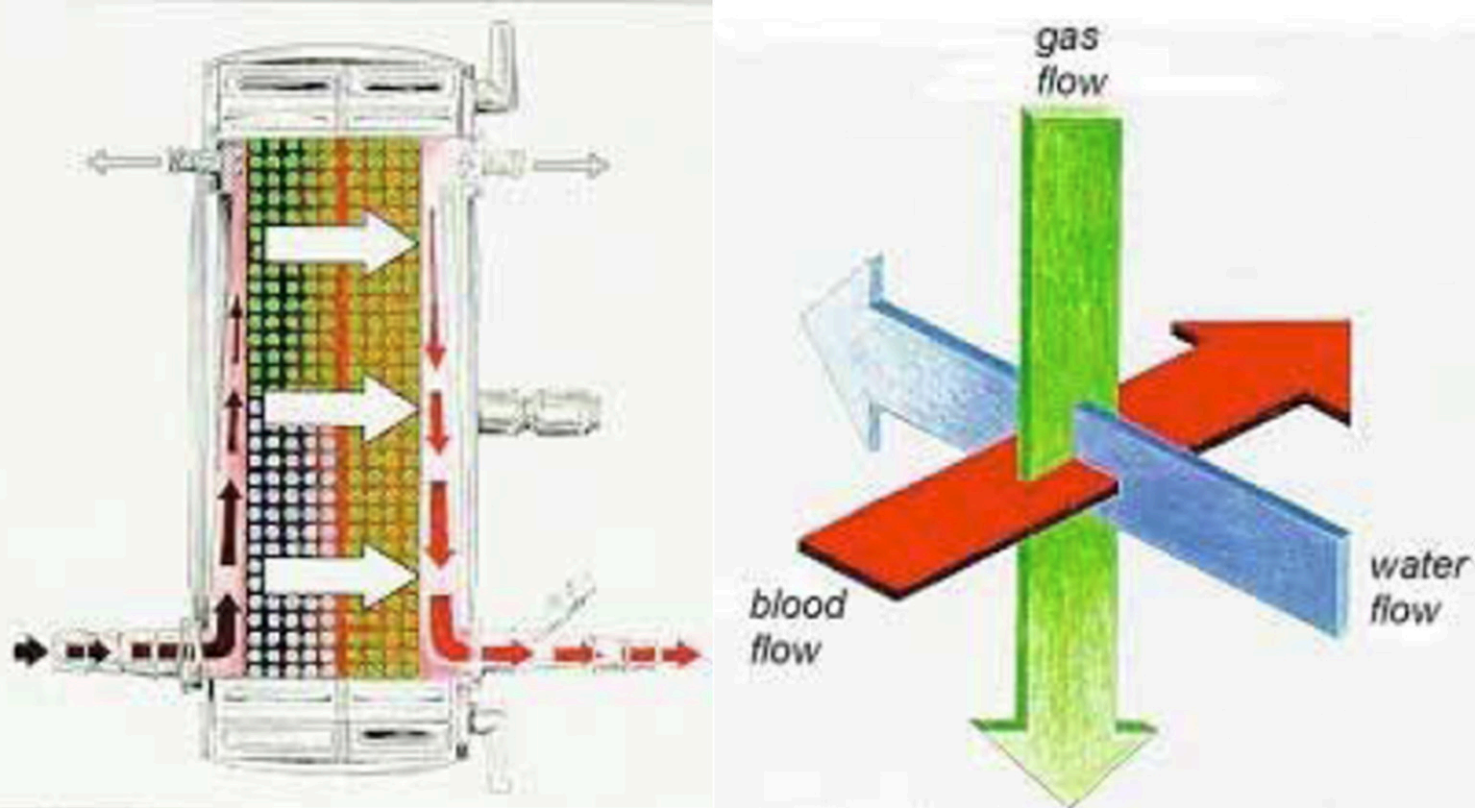
General flow path of blood through the Quadrox iD and NovaLung iLA devices (left). These devices employ an orthogonal flow path for the other medium (right). Blood flow is around the fibers while gas flow is through polymethylpentene fibers (Quadrox and NovaLung) and water flows through a separate set of polypropylene fibers (Quadrox only) in the right half of the oxygenator. The fibers are separated by a plastic divider (red vertical line in the left image).

In a PLA configuration, an oxygenator would need to present limited resistance to blood flow at the expected cardiac output. Short path oxygenators, where individual blood elements encounter as few oxygenator fibers as possible as they traverse the device, offer the ideal resistive profile (see Figure 1). This typically requires a larger surface area for equivalent gas exchange when compared to a long path oxygenator. The short path oxygenator configuration is often a cross-fiber mat, as opposed a long path oxygenator, which has a round, wound fiber configuration. To date, there are just two manufacturers for these types of oxygenators (see Table 1 for technical specifications on the available devices). These devices utilize a nearly identical design for their blood path (Figure 2). The primary difference between NovaLung iLA® (Xenios, Germany) and Quadrox® iD Adult (Maquet, Germany) is the absence of a mat of heat exchanger fibers in the iLA. This gives the iLA a thinner profile and a lower resistance to blood flow at 4 L/min (about half as much as the Quadrox iD Adult). For pediatric patients with an expected cardiac output less than 0.5 L/min, clinicians have used the Quadrox iD Pediatric (Maquet) with its incorporated heat exchanger (12). This oxygenator has been the primary polymethylpentene oxygenator used for pediatric ECMO in the United States for nearly a decade. However, it does have a sufficiently low resistance to permit its utilization in the PLA configuration in situations where the RV is capable of providing the driving pressure for blood flow.

**Table 1:** Technical specifications for oxygenators used in pumpless configuration.

**Manufacturer**	**Model**	**Pt Size**	**Surface area (m^2^)**	**Priming volume (mL)**	**VO_2_ (mL O_2_/min)**	**CO_2_ transfer rate (mL CO_2_/min)**	**Flow range (L/min)**	**ΔP at max flow (mmHg)**	**Connectors**	**Available coating**
Maquet	Quadrox® iD Pediatric	10 kg+	1.8	250	425	450	0.5-7	70	3/8″	BioLine (heparin), SoftLine (amphiphilic polymer)
Maquet	Quadrox® iD Neonatal	2–13 kg	0.8	81	180	140	0.25-1.5	38	1/4″	BioLine (heparin), SoftLine (amphiphilic polymer)
Xenios	NovaLung® iLA	10 kg+	1.3	175	130	148	0.5-4.5	20	3/8″	Heparin

### Clinical reports

Although there have been many reports of adults supported with PLA oxygenator technology for ECCO_2_R (13, 14) or as bridge to transplantation (15), there have been limited reports of its application in pediatric settings. Taylor et al. published the first case report of a 15-year old, 41 kg female with pulmonary veno-occlusive disease (16). This patient presented with a history of 6 months of increasing fatigue, dyspnea, and reduced exercise tolerance. She was diagnosed with veno-occlusive disease by echocardiographic findings of surprasystemic pulmonary artery pressures, and was managed with diuretics, warfarin and oxygen while being listed for transplantation. After three weeks, she deteriorated to the point of RV failure and hypoxemia, and was taken to the operating room for RV decompression using the NovaLung iLA. Due to increased risks associated with anesthesia in patients with pulmonary hypertension, she was prophylactically placed on VA ECMO through femoral cannulation under local anesthesia without sedation before undergoing a sternotomy under general anesthesia. Central cannulation was accomplished through the pulmonary artery (PA) using an arterial bypass cannula (22 FR EOPA, Medtronic, USA) and the right superior pulmonary vein using a right-angle single-stage bypass cannula (22 FR DLP, Medtronic). The patient was separated from ECMO after 100 min and maintained on the iLA device with flows of 1.8–2.2 L/min supported solely by the RV. She was extubated on postoperative day 3, had a device exchange on day 18 due to visible clot formation, and received a bilateral lung transplant on day 30 with discharge to home 25 days later. Her course with the iLA was uncomplicated, with no evidence of neurologic injury, and she was able to ambulate, breath spontaneously, and eat a normal diet. The authors reported that her hypertrophied RV returned to a normal size due to the afterload reduction offered by the iLA.

Gazit et al. published the first case report of the use of a PLA paracorporeal oxygenator in a small child (17). A 2-year-old male presented with pulmonary hypertension and was cannulated for VA ECMO through the neck on the second day of his hospital admission due to worsening hypoxia related to right to left shunting across the atrial septum. After 17 days of ECMO support, and listing for lung transplantation, the patient was transitioned to a NovaLung iLA through central cannulation of the PA with a 12 x 9-mm arterial Berlin Heart cannula (Berlin Heart, Germany). The LA was cannulated with a right-angle single-stage venous cardiopulmonary bypass cannula (22 FR, DLP, Medtronic). After 9 days on the iLA, the patient was extubated and allowed to mobilize. Periodic changes of the oxygenator were required for clot formation, and after 23 days of iLA support, the patient suffered a stroke; prompting removal of the iLA. Fortunately, during the period of iLA use, the dosing of pulmonary hypertensive medications (Bosentan and Flolan) were escalated and the patient demonstrated gradual decrease in the flow through his iLA as the pulmonary resistance declined. iLA flows went from 2.25 L/min at the initiation of support to 1.75 L/min at the time of device removal. The patient was discharged home, without transplantation, 3 months after initial presentation (~2 months after device removal).

Drawing on their initial success, Gazit et al. attempted to perform a similar rescue of a neonate with a diagnosis of alveolar capillary dysplasia (12). Again, with the intended endpoint of transplantation, the patient was transitioned to PLA support within 5 days of ECMO cannulation. Because the expected flow rate was less than the minimum 0.5 L/min of the iLA, a Quadrox iD Pediatric was used. A similar PA-LA cannulation was strategy was employed with a minor change to the PA cannulation in an attempt to achieve better hemostasis at this connection. The authors used a small 8-mm Gore-Tex (W.L.Gore Associates, Arizona, USA) graft anastomosed to the PA through which the 6 mm Berlin Heart atrial cannula was inserted. A 16 Fr right-angle venous cannula (DLP, Medtronic) was placed in the LA similar to the previous case. The initial flow rate was 0.25 L/min and increased to 0.45 L/min over the first 5 days. Anticoagulation was similar to their institutional ECMO protocol of continuous unfractionated heparin to maintain an ACT of 180–220 s. They added low-dose aspirin and antithrombin replacement to achieve antithrombin levels greater than 80%. Extubation occurred 15 days after device placement. The oxygenator and connectors were replaced on day 14 and 21, respectively due to thrombus build-up. Atrial ectopic tachycardia was noted and attributed to the LA cannulation, and a significant intracranial hemorrhage with seizure activity occurred on day 43, requiring redirection of care. The authors suggested that in light of seemingly appropriate anticoagulation strategy, better LA cannulation strategies might be required to mitigate the thrombogenic metal cannula tip in the LA, which they believe caused embolic strokes that led to the presentation of a hemorrhagic conversion stroke.

Another patient (2-month-old with AV canal with right lung hypoplasia and pulmonary interstitial glycogenesis) was treated with PLA as a bridge to lung transplant following placement on VA ECMO through the neck (18). This patient underwent AV canal repair at the time of device placement with the Quadrox iD Pediatric. Cannulation was similar to the prior neonatal patient, with the PA cannulated by a 6-mm Berlin Heart atrial cannula placed through an 8-mm Gore-Tex graft anastomosed to the PA, and a 16 Fr right-angle bypass cannula (DLP, Medtronic) placed in the LA. Anticoagulation was slightly lower in this older patient, compared to the prior neonate. Unfractionated heparin was administered with a target ACT of 160–200 s and antithrombin replacement to maintain levels greater than 80%. This patient's course was complicated by the presence of a small residual atrial septal defect, which led to a significant left to right shunt(Qp:Qs = 4:1) due to high left ventricular end-diastolic pressures from small left-sided structures that were not initially appreciated. Re-operation was required to address the shunt. Similar to the prior patients, this patient developed a hemorrhagic stroke by day 17. This event was speculated to be a hemorrhagic conversion of an embolic stroke originating from the LA cannulation strategy and resultant thrombus formation. The patient's course was further complicated by renal failure, leading to removal from the lung transplant list and redirection of care after 72 days of support.

The final case in the Gazit series reported on the successful bridge to transplant of a 9-month-old infant with pulmonary hypertension due to alveolar capillary dysplasia (18, 19). In this patient, transition from VA ECMO via neck vessels to PLA was also accomplished using a Quadrox iD Pediatric oxygenator connected between the PA and LA. The PA was directly cannulated with a 6-mm Berlin Heart atrial cannula because the patient was larger, with easier access to the PA. Due to the presence of strokes in the three prior patients, alternative LA cannulation was performed by placing a 10 mm Gore-Tex graft through the right atrium into an existing atrial septal defect (**Figure 4C**) to establish communication to the LA. The graft was then connected to a 6-mm Berlin Heart atrial cannula. Anticoagulation was maintained with unfractionated heparin to achieve ACTs between 160 and 200 s, but no antithrombin or aspirin was administered. Although the patient was only supported for 5 days before receiving a successful lung transplant, no thrombus was detected in the LA at the time of device explant. The authors hypothesized that this configuration stabilized the intracardiac portion of the cannula and improved outflow dynamics to the LA.

### Recommendations

The collective experiences of Gazit et al. have recently culminated in a series of recommendations for the use of PLA (7). The recommendations cover many aspects of pediatric patient management on this type of technology, including patient selection, contraindications, sedation, anticoagulation, and ventilator management.

Patient selection for PLA should be restricted to pulmonary hypertension (PH) for bridge to transplantation or bridge to recovery. The reason for this is that the resistance in the oxygenator must be less than the pulmonary vascular resistance to prevent blood preferentially flowing to the lungs, establishing a parallel blood path away from the lungs. In other forms of pulmonary failure where gas exchange is the primary mechanism, the PVR is not as elevated, and PLA would not improve the patient's condition without pulmonary artery banding distal to the PLA anastomosis. Because of the reliance of the heart to provide blood flow to the PLA, specific criteria for the cardiac function and anatomy need to be present. For patients with PH, there is evidence that the cardiac output is the strongest predictor for long-term survival (22). RV dysfunction, in addition to the sequelae from venous congestion (hepatic failure, ascites, reduced glomerular filtration rates), leads to LV dysfunction from lower filling pressures, decreased LV output, and decreased coronary perfusion. Cardiac failure can ultimately lead to the need for rescue decompression through VA ECMO, but may have irreversible fibrosis in the ventricles, for which PLA cannot provide relief. In fact, Gazit et al. have indicated that failure to wean from inotropic therapy in the initial days following PLA initiation may warrant further testing (echocardiographic and/or cardiac catheterization), and have suggested an ejection fraction <54% as a contraindication for PLA (7). In addition to global cardiac function, there are specific cardiac conditions that should be considered contraindications to PLA. With the low resistance from the PLA, atrial or ventricular level communications can result in a significant left atrial and left ventricular volume load. Therefore, any intracardiac communication, including single ventricle physiology, which results in clinically significant pulmonary overcirculation (18), should be considered a contraindication unless it can be repaired at the time of PLA implementation. This also includes greater than moderate mitral regurgitation, which would limit forward flow to the PLA.

Given the significant stroke rate (60%, 3/5 patients) reported in the early experience with PLA, sufficient anticoagulation and neurologic surveillance is necessary (16, 18). The surface area of PLA devices is the same as an ECMO oxygenator, and they require similar anticoagulation management with unfractionated heparin or direct thrombin inhibitors to achieve an activated partial thromboplastin time (aPTT) approximately two times the patient baseline value. With the oxygenator having a direct exit to the left atrium, the anticoagulation strategy may need to favor a more aggressive approach outside of the initial surgical period. Addition of aspirin to a heparin or direct thrombin inhibitor regimen may be appropriate without increasing bleeding risk (23). The most concerning areas for clot formation are the outlet of the oxygenator to the cannula returning to the left atrium. These areas should be monitored closely, and any clot formation on the outlet of the PLA system should be cause for concern and potential component change. The LA cannulation strategy may also be considered a significant factor in the reported strokes. The two patients who did not experience strokes had return cannulation through either, (1) an existing ASD using a Berlin Heart atrial cannula, or (2) through a pulmonary vein. Strategies to route blood to the LA while minimizing flow disturbances or stasis should be considered.

Monitoring of the PLA should include an ultrasonic flow probe and inlet pressure transducer. These two devices provide information about the relative state of the PLA device as well as potential remodeling in the pulmonary vasculature. Significant decreases in flow rate may be a result of thrombus build-up in the oxygenator, decrease in PVR permitting more blood to be directed to the lungs, or decrease in cardiac output. Careful examination of the patient and device are necessary to appropriately interpret these data. Thrombus build-up in the oxygenator may be associated with a decreased oxygen or CO_2_ transfer rate (difference in pO_2_ or pCO_2_ between inlet and outlet of the PLA device) or increased RV pressures in the setting of unchanged PVR. Decreased PVR relative to an unchanged device resistance should cause a decrease in RV pressures as blood is more easily shunted toward the lungs. Decreased cardiac output may indicate worsening cardiac function and should prompt an echocardiogram and hemodynamic assessment.

## Combined VAD and oxygenator technology

Pediatric pulmonary failure in conjunction with cardiac failure can present as a direct result of an anatomic or pathologic condition, or it can arise from the medical and surgical treatment of these conditions. An acute presentation of biventricular failure or cardiorespiratory failure typically leads to the utilization of traditional ECMO strategies because of physician familiarity of these systems and efficiency of deployment (24, 25). On ECMO, a patient with severe ventricular failure and no intracardiac shunt may require the addition of a left atrial or left ventricular vent to prevent harmful distension of the left ventricle (26). In the case of isolated poor left (or systemic) ventricular function or non-acute presentation, VAD support with adequate decompression of the failing ventricle is central to a strategy for myocardial recovery. Therefore, starting with a short-term paracorporeal LVAD or quickly transitioning from ECMO to a short-term LVAD within a few days may provide the necessary decompression without additional cannulae or an atrial septostomy via cardiac catheterization (27).

Conversion to VAD systems typically occurs after several days of traditional ECMO when: (1) the cause of the acute decompensation is determined, (2) end-organs have been resuscitated, and (3) the expected recovery time is lengthy or transplantation is the ultimate goal. Bridge to recovery occurs relatively infrequently (1–11%) in adult VAD patients (28, 29), and appears to be more frequent (35–83%) in pediatric VAD patients when the appropriate etiology is recognized (24–27, 30). Of all heart conditions that lead to implementation of mechanical circulatory support, post-partum cardiomyopathy (adults) and myocarditis (children) have the highest rates of recovery, respectively (26, 28, 30, 31). Despite optimal medical management on a VAD, severe pulmonary failure may still occur; leaving clinicians with the difficult choice to return to ECMO, or to create a hybrid VAD+Oxy system to support the patient through this pulmonary failure phase (32–34). This section describes the collective experience of those who have chosen the latter for their patients, and insights gained from their efforts.

### Paracorporeal VADs used with oxygenators

The lack of intracorporeal VADs labeled for patients with a BSA <1.2 m^2^ has spurred the use of adult or pediatric paracorporeal VADs as a bridge to transplantation or a bridge to recovery. The ability to provide short-term oxygenator support in the process is technically feasible because of the externalization of the VAD. The choice of device has largely depended on institutional resources and surgeon preference or comfort level with the technology. Paracorporeal VADs fall into two broad categories based on their blood flow characteristics: continuous or pulsatile.

**Figure 3:**
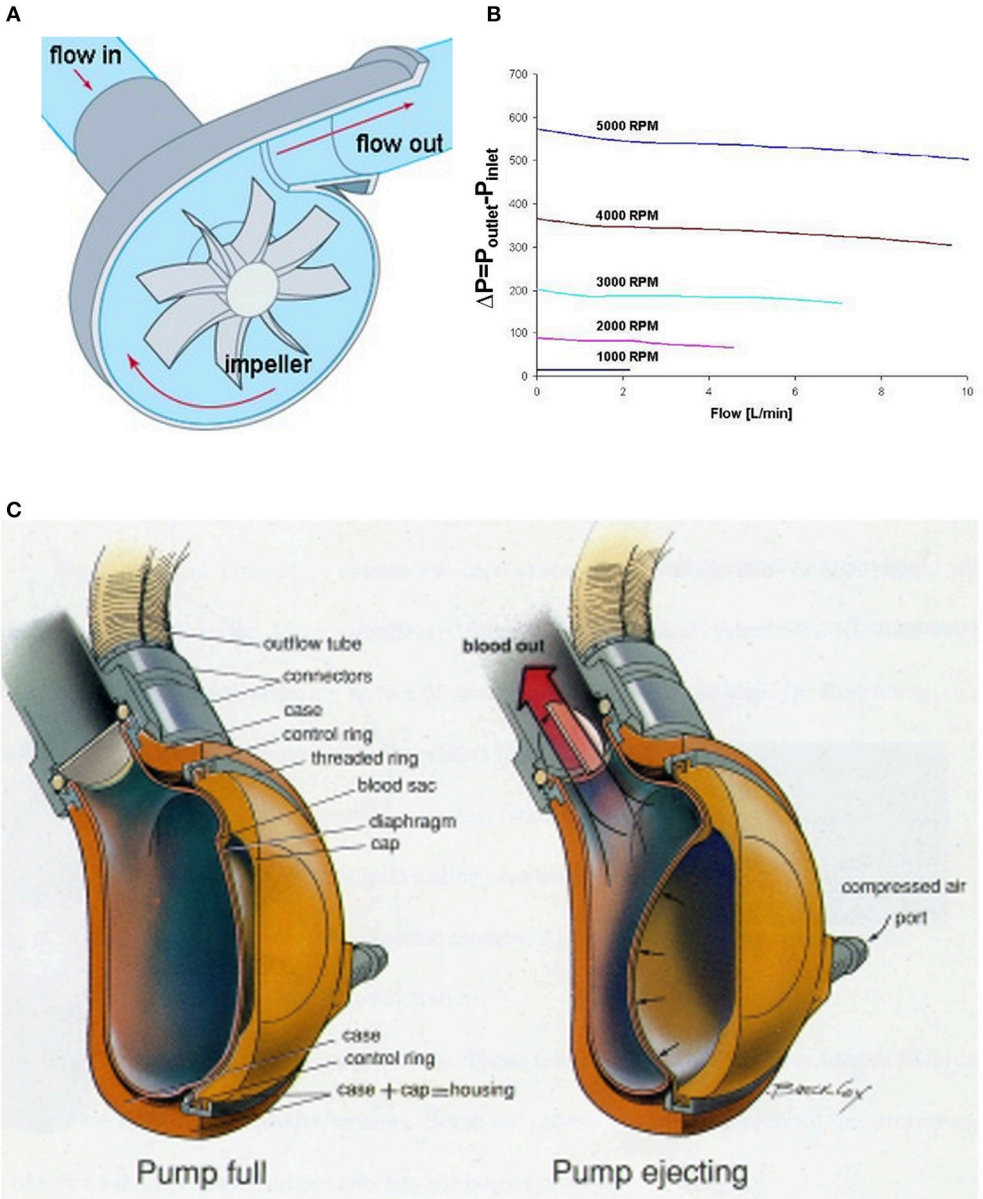
VADs used in VAD+Oxy configurations. **(A)** In a centrifugal pump, blood flow is directed inward from the inlet, accelerated circumferentially by the impeller, and then expelled along the axial line of the outlet. **(B)** The pressure change across a typical centrifugal pump is fixed by the impeller speed. The resultant flow is therefore a function of the inlet and outlet resistance to flow. **(C)** A pneumatically driven paracorporeal VAD has an internal blood-filled sac compressed externally by air forced between it and the housing. One-way valves create unidirectional flow from the pump. Adapted with permission from ASME (35).

Extracorporeal continuous flow VADs are generally considered centrifugal pumps because their inlet and outlet ports from the device are orthogonal to each other and the primary motion of blood in the device is circumferential until it exits the outlet flute. A typical centrifugal pump is shown in Figure 3A. Centrifugal pumps are pressure-generating pumps because the action of the rotating impeller creates a specific pressure difference between the inlet and outlet of the pump. Consequently, for a specific number of revolutions per minute (RPM) of the pump, the actual blood flow may vary depending upon the resistance to flow at either the inlet (preload) or outlet (afterload) from the pump (see Figure 3B). The paracorporeal centrifugal pumps used for VAD+Oxy configurations have included the PediMag® and CentriMag® (Abbott, California, USA), Rotaflow® (Maquet, Germany), and TandemHeart® (Cardiac Assist Technology, Pennsylvania, USA). The key differences between these pumps are in their sizes, specific pressure profiles (the amount of pressure generated for a given RPM) and the type of bearing that allows the impeller to spin within the housing (see Table 2).

**Table 2:** Listing of devices and technical specifications for pumps used in reports of VAD+Oxy configurations.

**Company**	**Device**	**Type**	**Priming volume (mL)**	**Flow range (L/min)**	**Key features**	**Connection**
Abbott	CentriMag®	Continuous flow, Centrifugal	32	1–8	Magnetically Levitated Impeller	3/8″
Abbott	PediMag®	Continuous flow, Centrifugal	14	0.2–1.8	Magnetically Levitated Impeller	1/4″
Cardiac Assist	TandemHeart®	Continuous flow, Centrifugal	10	1–4	Liquid cooled bearing	3/8″
Maquet	RotaFlow®	Continuous flow, Centrifugal	32	1–10	Hydrodynamic Bearing	3/8″
Berlin Heart	Excor®	Pulsatile, Pneumatic	10, 15, 25, 30, 50, 60	0.3–7.5	Tri-leaflet valves	1/4″-3/8″
Medtronic	AB5000®	Pulsatile, Pneumatic	80	2–6	Tri-leaflet valves	3/8″

Pulsatile VADs in the paracorporeal setting are pneumatically driven devices with blood-filled polymer sacs enclosed in hard external housings (Figure 3C). Filling and emptying of the blood sac is controlled through the applied pneumatic pressure and vacuum settings, the timing of systole and diastole, and preload and afterload resistances. One-way valves positioned at the entrance and exit of the blood sac create unidirectional flow through the device. Although there is some sensitivity to preload and afterload resistance, these pumps are less sensitive to these changes than their centrifugal counterparts. Key areas of concern are the valves, which are sources of thrombus formation. To date, only two paracorporeal pneumatically driven VADs have been reported with oxygenators spliced in-line: the EXCOR® (Berlin Heart) and AB5000® (Abiomed, Massachusetts, USA).

Cannulation for an LVAD is typically from the LA or LV (depending on patient size, pathologic substrate, and available cannula options) to the aorta. Cannulation for an RVAD is typically from the RA to the PA. Cannulation of the right atrium may be accomplished with a right-angle cannula or a Berlin Heart atrial cannula. Cannulation of the ventricle is typically accomplished with a straight cannula or a Berlin Heart ventricular cannula. Decisions for cannula type and position are based upon patient anatomy, patient size, and availability of the cannula (e.g., centers that do not use the Berlin Heart as a VAD typically do not have access to Berlin Heart cannulae). Outflow cannulae typically consist of Berlin Heart cannulae or a vascular graft (Dacron or Gore-Tex) anastomosed to the aortic or pulmonary artery for an LVAD or RVAD, respectively (36). Because of the variety of devices, cannulae and tubing, few patients are provided with tip-to-tip coating with anti-coagulant or anti-inflammatory materials that are typically found in modern ECMO systems.

### Clinical reports

Garcia-Guereta et al. were among the first to report the use of an oxygenator in series with a VAD in children (37). They reported a case report of a 5-year-old with acute onset heart failure from myocarditis. After a worsening clinical course, the patient was listed for heart transplantation and subsequently experienced a cerebrovascular accident from an LV thrombus and further deterioration of LV function. The patient was placed on ECMO for 9 days prior to conversion to Berlin Heart biVADs. Hemorrhagic atelectasis in the operating room during biVAD placement required additional oxygenator support, which was placed in the outflow of the left pump. The oxygenator was weaned after 3 days, and the patient extubated 2 weeks later. After 210 days, the patient was successfully transplanted.

**Figure 4:**
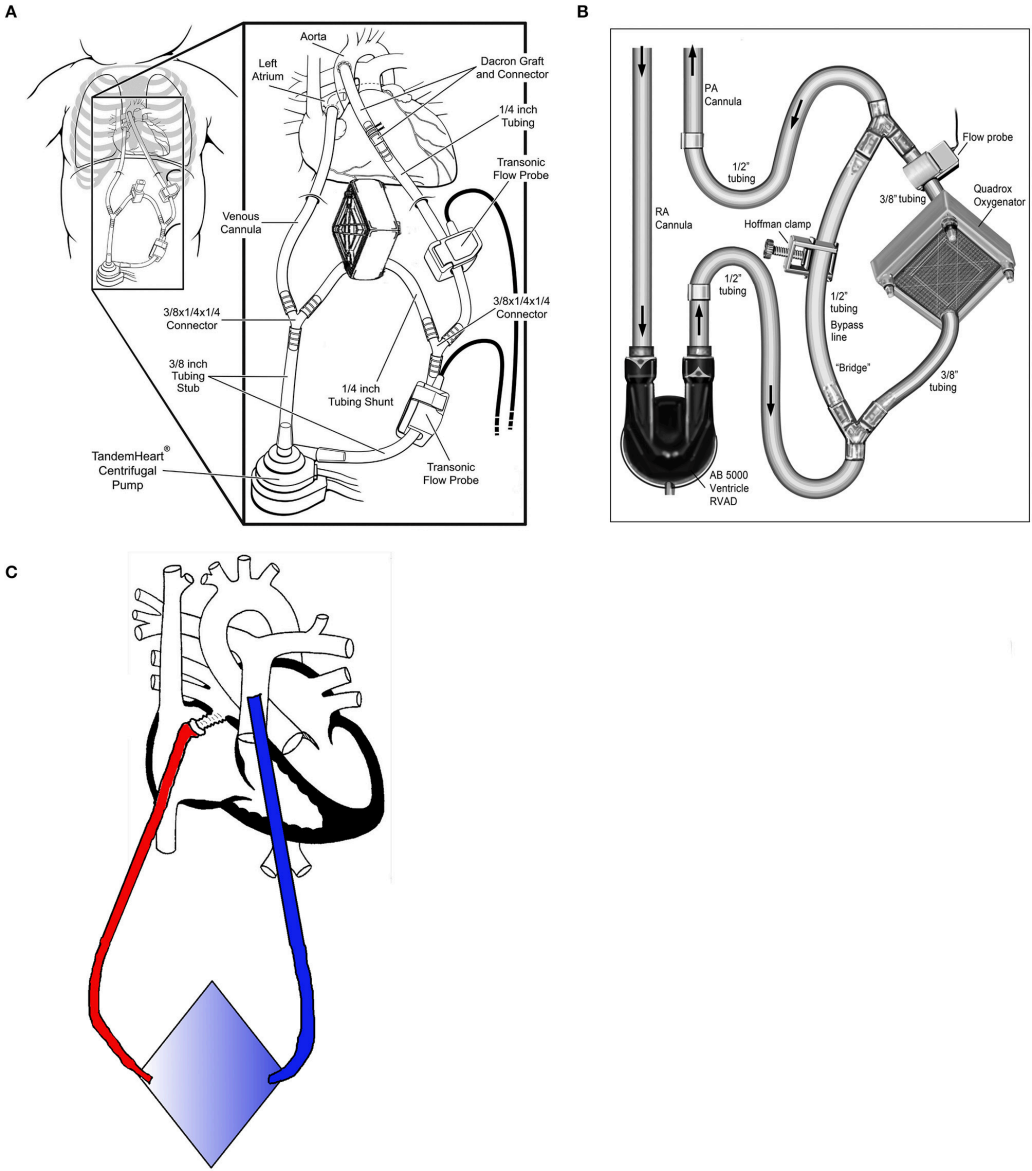
Configurations for paracorporeal oxygenators. **(A)** Centrifugal pump with oxygenator in the shunt line [modified with permission from Annals of Thoracic Surgery (20)]. **(B)** Pulsatile pump with oxygenator and shunt line [reproduced with permission from the Journal of Extracorporeal Technology (21)]. **(C)** Pumpless oxygenator configuration with return through ASD.

Betit et al. reported the use of a VAD+Oxy in a single patient with acute respiratory distress (21). During the post-operative period following a root replacement in a 12-year-old with Marfan syndrome, AB5000 biVADs were placed. The patient developed increasing ventilator requirements, and a Quadrox oxygenator was spliced into the outlet of the RVAD. Because the patient was dependent upon the biVAD for cardiac output, the authors constructed their circuit with a bridge around the oxygenator to facilitate a potential change in oxygenators without interrupting RVAD flow (Figure 4B).

In 2017, Monge et al. reported a small case series utilizing an adult paracorporeal continuous-flow VAD (Tandem Heart, Cardiac Assist, Pittsburgh, USA) in 13 pediatric patients (0.4–2.1 m^2^ BSA), including three patients with single ventricle physiology (20, 38). All patients had their left atrium (or common atrium for single ventricles) cannulated using a standard metal-tip right-angle bypass cannula. A Gelweave Dacron graft (Terumo, Ann Arbor, USA) was anastomosed to the ascending aorta for outflow. The Tandem Heart is a small centrifugal pump (priming volume of ~10 mL) but has a minimum flow rate of 1.0 L/min, which is too high for children <0.8 m^2^, and requires a recirculation line. Three patients required an oxygenator spliced into outlet tubing from the pump, with two being placed in the recirculation line (Figure 4A). Twelve of the patients in the series underwent transplantation, including those requiring additional oxygenators. One patient recovered cardiac function without transplant. The average support time was 31 days for the cohort, regardless of the use of the oxygenator. The authors cited the stroke rate of EXCOR (11–45%) as a primary reason why they opted for adult centrifugal technology. In their series, 2 of 13 patients (14%) had strokes. While this was significantly better than the worst reported stroke rate for the EXCOR, it does not appear to be any better than the most recent experiences where anticoagulation management and selection of EXCOR size has greatly improved.

Zacagni et al. reported the use of an oxygenator with EXCOR biVADs to support an 11-month-old girl with chronic severe heart failure secondary to a complications of a neonatal arterial switch operation (39). This patient was transitioned from ECMO to biventricular EXCOR pumps. Three weeks after transition to bi-VAD, she developed pulmonary hemorrhage, hypercarbia and associated pulmonary hypertension that caused complete cardiovascular collapse. A pediatric Quadrox oxygenator was spliced into the RVAD circuit between the blood pump and the pulmonary artery. She was maintained on a Berlin Heart anticoagulation regimen (warfarin and aspirin) in addition to 10 IU/kg/h heparin infused into the inlet of the oxygenator. The oxygenator remained functional for 2 weeks and was removed, and the patient was subsequently extubated.

Conway et al. published their institutional experience with short-term continuous flow devices (CentriMag, PediMag, and Rotaflow) for 27 patients with pediatric cardiac failure in 2016 (27). The patient population included 14 with congenital heart defects (including 3 single ventricle patients), 11 with cardiomyopathy, and 6 post heart transplant. Twenty of the 33 reported VAD runs (61%) required additional oxygenator support placed in series with the paracorporeal VAD. Seven of the patients required biventricular VAD placement (2 paracorporeal VADs), and all but one of those patients required oxygenator support. The oxygenator was placed in series with the RVAD in these patients in order to provide oxygenated blood to the pulmonary arteries (known to decrease pulmonary vascular resistance) and to reduce stroke risk from microemboli originating on the large surface area of the oxygenator. RVADs were the least commonly used modality (8 RVADs in 5 patients), and 5 of the 8 RVADs (63%) required an addition of an oxygenator. Fifteen of the 20 VAD runs (75%) requiring an oxygenator were on ECMO in the 24 h prior to VAD implantation, which indicates a significant degree of lung failure early in the disease course. The authors' experience regarding the level of oxygenator support required for VAD patients is greater than reported by others. One previous single-center reported conversion from mechanical circulatory support for heart transplantation to ECMO in about 23% of patients (40). The PEDIMACS registry of pediatric VADs reported only 7% of their patients had prior ECMO runs, and 13% had respiratory failure within 3 months of implantation (41). Overall outcomes were positive, with only 5 patients (19%) expiring on support or within 1 month of decannulation, which is better than the 33% mortality reported in the ELSO registry for pediatric cardiac ECMO (1).

Nelson-McMillan et al. most recently reported the use of an oxygenator in a 7-year-old patient who acquired a respiratory virus and required high frequency oscillatory ventilation (HFOV) following EXCOR biVAD implantation for presumed myocarditis (42). On post-operative day 24, the patient developed a fever and an elevated C-reactive protein that was attributed to cytomegalovirus (CMV) infection. After optimizing ventilator support, the LVAD filling was <10% and there was minimal aortic valve opening. On day 34, a Quadrox D was placed in series with the RVAD to facilitate lung rest with lower ventilator settings, and, as a result, improve filling of the LVAD. The patient was maintained on an unfractionated heparin infusion, targeting anti-Xa and aPTT values of 0.5 IU/mL and aPTT 60–80 s. After 10 days, the RVAD and oxygenator were replaced due to hemodynamically significant RVAD valve incompetence. The authors believed the valve failure was caused by prolonged exposure to the additional resistance from the oxygenator. The oxygenator was removed after 19 days, but the patient developed septic shock 1 month later (requiring the oxygenator to be placed in-line again) and subsequently died from multi-organ failure.

### Recommendations

The addition of an oxygenator to a paracorporeal VAD would appear to potentially be a relatively straightforward process. There are many reasons for adding an oxygenator to a paracorporeal VAD rather than initiating separate ECMO support, or instituting ECMO in the place of the VAD. Avoidance of additional surgical procedures, such as neck or groin cannulation for ECMO, or surgical removal of VAD cannula, minimizes short-term procedural risks, as well as long-term complications such as femoral vein and artery thrombosis related to vessel manipulation. Avoiding simultaneous management of two complex mechanical circulatory support systems is also preferable. The published experience with VAD+Oxy configurations demonstrates both frequent use and reasonably good short-term outcomes. However, much like PLA technology, VAD+Oxy configurations should be reserved for a specific subset of patients, and managed by an experienced team. In certain contexts, there may be no actual benefit conferred to the patient by utilizing VAD+Oxy configurations compared to multiple combined modalities (40).

Key considerations for initiating VAD+Oxy are, (1) Patient age and size; (2) disease process; (3) anticoagulation; and (4) the capabilities and comfort of the center in managing non-traditional systems. Because younger and smaller patients (<10 kg) tend to have a higher incidence of pulmonary failure and biventricular failure, they are more likely to require multiple modalities in their bridge-to-transplantation process, compared to their older pediatric counterparts (40, 43). Also, younger patients need smaller devices, which have lower margins for error and are not well-equipped for overcoming high resistances. These limitations are particularly important among the pneumatic devices. The disease process also plays a key role in decision-making. Primary respiratory failure due to viral illness or pulmonary edema may be easily managed with a VAD+Oxy configuration, while bacterial sepsis involving multi-organ dysfunction and cardiovascular instability will benefit from the control and vascular access afforded by conversion to VA ECMO (27). Anticoagulation on a VAD+Oxy patient is primarily dependent left- or right-sided support and mirrors that provided to ECMO patients because of the additional surface area of the oxygenator. Anticoagulation for left-sided support will most closely resemble that for a chest-cannulated VA ECMO patient. Early anticoagulation will be minimal to permit surgical hemostasis, followed by a more aggressive anticoagulation strategy that should include anti-platelet agents to prevent thrombotic stroke. Anticoagulation on right-sided support will most closely resemble that for VV ECMO, which can be less aggressive because of the additional filtration protection afforded by the lungs. Finally, one of the most important considerations is the experience level of the medical management team, including device first-responders such as bedside nurses, perfusionists, respiratory therapists, and/or ECMO/VAD specialists. Teams that are comfortable with the day-to-day management of ECMO patients are likely to be successful with VAD+Oxy configurations because personnel are well equipped to troubleshoot technical issues with the devices, and to recognize and respond to emergencies.

## CO_2_ removal devices for pediatric ECMO

In addition to the increased interest in VV ECMO for acute respiratory failure, several small, easy to use devices have entered the marketplace specifically targeting patient with chronic obstructive pulmonary disease (COPD) and cystic fibrosis. Designed for institutions without active ECMO programs, these ECCO_2_R devices are essentially small oxygenators and blood pumps that come as an integrated device or cartridge and connect to the patient utilizing small catheters (similar in size to dialysis catheters) that can be easily placed percutaneously in the intensive care unit. The core principle is that at low blood flow rates, a high sweep rate on a low surface area device can remove sufficient CO_2_ from the patient's blood to improve their condition. Results have been promising, and some manufacturers are exploring whether these devices could be used instead of invasive mechanical ventilation. The technical aspects of each of the three devices that have been utilized for ECCO_2_R may also make them suitable for other applications such as a pediatric ECMO platform.

### Devices

**Figure 5:**
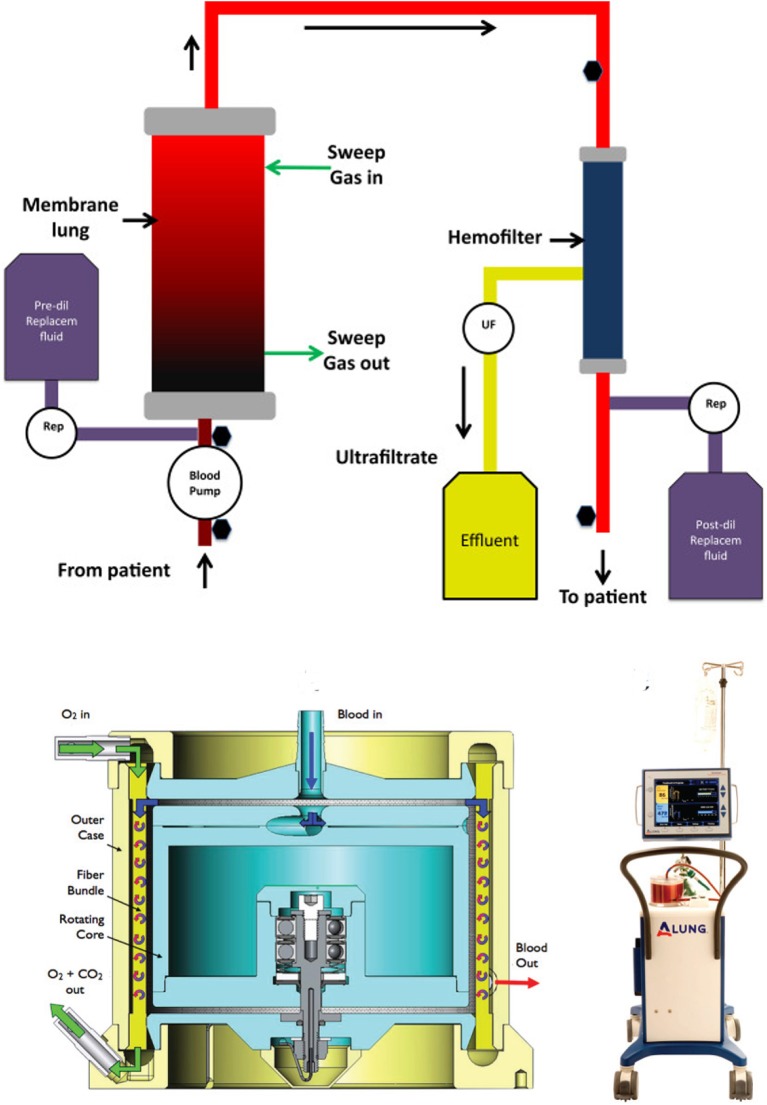
Graphical representation of an ECCO_2_R device with renal support (top). The oxygenator is typically placed in series with the hemofilter to increase the resistance through the oxygenator and reduce the chance for bubble formation. The HemoLung (bottom) is an out-of-the-box ECCO_2_R device with a unique active mixing feature to improve mass transfer at the fiber surface.

A number of devices are in development or have been marketed for ECCO_2_R in Europe (see Table 3) simply replace a smaller oxygenator in an ECMO system (iLA Active® and PALP^TM^) or add an oxygenator to an existing hemodialysis/continuous renal replacement therapy system (Abylcap®, Prisma-Lung®, Aferetica®, see Figure 5 top) (44, 45). The oxygenator sizes range from 0.3 m^2^ to nearly 1.0 m^2^ with flow rates ranging from the ultra-low (30 mL/min) to traditional ECMO flows (4.5 L/min). These sizes and flow ranges seem to be perfectly suited for ECMO in the pediatric population, and have the added benefit of incorporated renal replacement therapy or cytokine filtration (Abylcap®), which may be necessary for patients presenting with sepsis in addition to pulmonary failure.

**Table 3:** ECCO_2_R devices currently marketed or in development.

**Company**	**Device**	**Marketing**	**Features**
Xenios	Novalung iLA active®	ECCO_2_R to ECMO	Small diagonal pump (0.5–4.5L/min) in a portable console with the iLA oxygenator
Maquet	PALP®	ECCO_2_R	Low-flow system (0.2–2.8 L/min) based on CardioHelp platform and using a small (0.98 m^2^) oxygenator
A-Lung technologies	HemoLung®	ECCO_2_R	Small surface area (0.67 m^2^) membrane lung with active mixing to improve diffusion
Medtronic	Abylcap®	ECCO_2_R and sepsis/renal support	Small membrane (0.67 m^2^) and low flow (0.28-0.35 L/min) inserted into the Lynda® coupled plasma filtration system
Medos	Prisma-Lung®	ECCO_2_R and renal support	Small membrane (0.32 m^2^), low flow (0.45 L/min) oxygenator added into the Prisma® hemodialysis system
Aferetica purification therapy	Aferetica®	ERRO_2_R and renal support	Low flow (0.03–0.45 L/min) oxygenator (unspecified surface area) inserted into hemodialysis system

Of the ECCO_2_R devices, the HemoLung® is the only one to provide a truly a unique design for its oxygenator. The HemoLung incorporates the pump and oxygenator into the same unit by spinning a hollow fiber oxygenator bundle. This creates a disturbed boundary layer (marketed as “active mixing”) at the fiber surface and improves gas transfer (Figure 5 bottom) for a given size oxygenator, allowing the device to become more compact. HemoLung is also the only device that has been evaluated for its applicability to a pediatric ECMO population. Jeffries et al. published promising preclinical studies in a juvenile ovine model (46). In that study, a series of 12 animals (19-33 kg) underwent 1-week ECMO runs using the HemoLung as the oxygenator with flow rates sufficient for a 3 kg neonate. Seven animals successfully reached study conclusion. The remaining five animals were terminated early due to complications with central venous catheters and monitoring lines. No device failures occurred. Circuit blood flow was maintained at 280–300 mL/min for the duration of the study, resulting in 50 mL/min CO_2_ removal. Blood drawn from the outlet of the HemoLung was 100% saturated with oxygen for the duration of all studies. These data, along with bench testing demonstrating 0.3–0.8 L/min blood flow, led the authors to conclude that for a specific pediatric application (3–10 kg), the HemoLung could sufficiently transfer enough oxygen to serve as a VV ECMO device. Since the device is a self-contained, cartridge-style system, it is primarily marketed toward low-volume centers where technical training is limited and more user-friendly solutions, requiring fewer employees to manage may be desirable. This is a similar approach to the design of the CardioHelp® (Maquet), which was designed as a more self-contained, cartridge style, rapidly deployable ECMO system.

## Conclusions

The utilization of ECMO in non-traditional clinical situations has been reported in case reports and small case series. To date, anecdotes of these off-label applications of ECMO and VAD technology from the field indicate that additional paracorporeal oxygenator support with or without a paracorporeal VAD can be accomplished by experienced practitioners. These approaches may ultimately become valuable tools in a clinician's armamentarium as they bridge their patients to transplantation. Although experience with these devices has greatly improved the duration that a patient may be safely supported, paracorporeal technology (both VADs and oxygenators), at this time, is not suitable as a destination therapy. Key takeaways from these experiences are: (1) an appreciation for the complex interplay between the underlying anatomy and physiology of the patient and the technical attributes of the available paracorporeal devices, and (2) the importance of minimizing the risk of thromboembolic events by using a customized cannulation and anticoagulation strategy. Anticoagulation regimens vary between institutions, and can vary within an institution's active ECMO program on a patient to patient basis or depending on the type of ECMO [veno-venous (VV) or veno-arterial (VA)]. Given the nature of PLA, strategies for anticoagulation that are more in alignment with VA ECMO, where the stroke risk is much higher than VV ECMO, should be considered. Specific anticoagulation strategies are outside the scope of this review, but several excellent reviews have been published recently that can help guide practitioners in their decision-making (47–49). In the authors' opinion, one of the most important aspects of anticoagulation is to have a written protocol, which surprisingly over 25% of ECMO centers do not currently have (50). These reports also highlight the ingenuity and resourcefulness of experienced programs who utilize ECLS technology on a daily basis. There is a clear need for more advanced and specific technology in pediatrics.

Although the initial reports from the pioneers in this field are encouraging, collective experience with paracorporeal lung devices to date is limited. Much like the early ECMO experiences, a few positive case reports followed by widespread application is likely to result in significant complications and undesirable outcomes. These case reports have already highlighted areas of significant concern, particularly the stroke risk associated with PLA technology that could benefit from additional research and technological advancement. Case reports can also overlook details that may prove crucial toward determination of adequate indications and contraindications. Data registries can provide a central location to examine the collective experience of a field for this purpose. Thanks to the efforts of the Extracorporeal Life Support Organization (ELSO) Registry over the past 30 years, we have even begun to expand the indications and windows for recovery; 2 weeks is no longer the time limit for ECMO, and sepsis or congenital cardiac heart disease is no longer a contraindication. However, current registries are not adequately equipped to capture these cases without revision to be more flexible in their description of the technology application or to capture conversions between therapies. Registry intercommunication and more longitudinal data capture may provide a better understanding of the size of this particular patient population and the benefits and complications of these approaches.

## Author contributions

TM contributed initial drafting of the chapter. JN and PW provided editorial support and made significant revisions to the chapter.

### Conflict of interest statement

The authors declare that the research was conducted in the absence of any commercial or financial relationships that could be construed as a potential conflict of interest.

## References

[1] ECLS Registry Report: US Summary Ann Arbor, MI: extracorporeal Life Support Organization (2017).

[2] Gadepalli SK Hirschl RB. Extracorporeal life support: updates and controversies. Semin Pediatr Surg. (2015) 24:8–11. 10.1053/j.sempedsurg.2014.11.002 25639803

[3] Rais-Bahrami K Van Meurs KP. Venoarterial versus venovenous ECMO for neonatal respiratory failure. Semin Perinatol. (2014) 38:71–7. 10.1053/j.semperi.2013.11.003 24580762

[4] Cooper DS Jacobs JP Moore L Stock A Gaynor JW Chancy T. Cardiac extracorporeal life support: state of the art in 2007. Cardiol Young. (2007) 17(Suppl. 2):104–15. 10.1017/S1047951107001217 18039404

[5] Puri V Epstein D Raithel SC Gandhi SK Sweet SC Faro A. Extracorporeal membrane oxygenation in pediatric lung transplantation. J Thorac Cardiovasc Surg. (2010) 140:427–32. 10.1016/j.jtcvs.2010.04.012 20538306

[6] Lehr CJ Zaas DW Cheifetz IM Turner DA. Ambulatory extracorporeal membrane oxygenation as a bridge to lung transplantation: walking while waiting. Chest (2015) 147:1213–8. 10.1378/chest.14-2188 25940249

[7] Gazit AZ Sweet SC Grady RM Boston US Huddleston CB Hoganson DM. Recommendations for utilization of the paracorporeal lung assist device in neonates and young children with pulmonary hypertension. Pediatr Transplant. (2016) 20:256–70. 10.1111/petr.12673 26899454

[8] Castagnola E Gargiullo L Loy A Tatarelli P Caviglia I Bandettini R. Epidemiology of infectious complications during extracorporeal membrane oxygenation in children: a single-center experience in 46 runs. Pediatr Infect Dis J. (2018) 37:624–6. 10.1097/INF.0000000000001873 29278612

[9] Thomas J Kostousov V Teruya J. Bleeding and thrombotic complications in the use of extracorporeal membrane oxygenation. Semin Thromb Hemost. (2018) 44:20–9. 10.1055/s-0037-1606179 28898902

[10] Murphy DA Hockings LE Andrews RK Aubron C Gardiner EE Pellegrino VA. Extracorporeal membrane oxygenation-hemostatic complications. Transfus Med Rev. (2015) 29:90–101. 10.1016/j.tmrv.2014.12.001 25595476

[11] Kawamura T Fukui Y. Extracorporeal membrane oxygenation (ECMO) in pumpless right ventricle to left atrium bypass. Trans Am Soc Artif Intern Organs. (1985) 31:616–21. 3837520

[12] Hoganson DM Gazit AZ Sweet SC Grady RM Huddleston CB Eghtesady P. Neonatal paracorporeal lung assist device for respiratory failure. Ann Thorac Surg. (2013) 95:692–4. 10.1016/j.athoracsur.2012.05.128 23336880

[13] Hammell C Forrest M Barrett P. Clinical experience with a pumpless extracorporeal lung assist device. Anaesthesia. (2008) 63:1241–4. 10.1111/j.1365-2044.2008.05582.x 18717661

[14] Walles T. Clinical experience with the iLA Membrane Ventilator pumpless extracorporeal lung-assist device. Expert Rev Med Devices. (2007) 4:297–305. 10.1586/17434440.4.3.297 17488224

[15] Fischer S Hoeper MM Bein T Simon AR Gottlieb J Wisser W. Interventional lung assist: a new concept of protective ventilation in bridge to lung transplantation. ASAIO J. (2008) 54:3–10. 10.1097/MAT.0b013e318161d6ec 18204308

[16] Taylor K Holtby H. Emergency interventional lung assist for pulmonary hypertension. Anesth Analg. (2009) 109:382–5. 10.1213/ane.0b013e3181ac5461 19608807

[17] Gazit AZ Sweet SC Grady RM Huddleston CB. First experience with a paracorporeal artificial lung in a small child with pulmonary hypertension. J Thorac Cardiovasc Surg. (2011) 141:e48–50. 10.1016/j.jtcvs.2011.02.005 21420104

[18] Hoganson DM Gazit AZ Boston US Sweet SC Grady RM Huddleston CB. Paracorporeal lung assist devices as a bridge to recovery or lung transplantation in neonates and young children. J Thorac Cardiovasc Surg. (2014) 147:420–6. 10.1016/j.jtcvs.2013.08.078 24199759

[19] Boston US Fehr J Gazit AZ Eghtesady P. Paracorporeal lung assist device: an innovative surgical strategy for bridging to lung transplant in an infant with severe pulmonary hypertension caused by alveolar capillary dysplasia. J Thorac Cardiovasc Surg. (2013) 146:e42–3. 10.1016/j.jtcvs.2013.06.014 23871141

[20] Monge MC Kulat BT Eltayeb O Balasubramanya S Sarwark AE Zingle NR. Novel modifications of a ventricular assist device for infants and children. Ann Thorac Surg. (2016) 102:147–53. 10.1016/j.athoracsur.2016.04.043 27240450

[21] Betit P Matte GS Howe R Iudiciani P Barrett C Thiagarajan R. The addition of a membrane oxygenator to a ventricular assist device in a patient with acute respiratory distress syndrome. J Extra Corpor Technol. (2011) 43:264–6. 22416609 PMC4557432

[22] Kula S Pektas A. A review of pediatric pulmonary hypertension with new guidelines. Turk J Med Sci. (2017) 47:375–80. 10.3906/sag-1605-172 28425226

[23] Bein T Zimmermann M Philipp A Ramming M Sinner B Schmid C. Addition of acetylsalicylic acid to heparin for anticoagulation management during pumpless extracorporeal lung assist. ASAIO J. (2011) 57:164–8. 10.1097/MAT.0b013e318213f9e0 21427564

[24] Jefferies JL Morales DL. Mechanical circulatory support in children: bridge to transplant versus recovery. Curr Heart Fail Rep. (2012) 9:236–43. 10.1007/s11897-012-0103-y 22805892

[25] Wilmot I Morales DLS Price JF Rossano JW Kim JJ Decker JA. Effectiveness of mechanical circulatory support in children with acute fulminant and persistent *Myocarditis*. J Card Fail. (2011) 17:487–94. 10.1016/j.cardfail.2011.02.008 21624737

[26] Adachi I Burki S Zafar F Morales DLS. Pediatric ventricular assist devices. J Thoracic Dis. (2015) 7:2194–202. 10.3978/j.issn.2072-1439.2015.12.61 26793341 PMC4703653

[27] Conway J Al-Aklabi M Granoski D Islam S Ryerson L Anand V. Supporting pediatric patients with short-term continuous-flow devices. J Heart Lung Transplant. (2016) 35:603–9. 10.1016/j.healun.2016.01.1224 27009672

[28] Topkara VK Garan AR Fine B Godier-Furnémont AF Breskin A Cagliostro B. Myocardial recovery in patients receiving contemporary left ventricular assist devices: results from the Interagency Registry for Mechanically Assisted Circulatory Support (INTERMACS). Circ. Heart Fail. (2016) 9:e003157. 10.1161/CIRCHEARTFAILURE.116.003157 27402861 PMC4943678

[29] Kirklin JK Naftel DC Pagani FD Kormos RL Stevenson LW Blume ED. Seventh INTERMACS annual report: 15,000 patients and counting. J Heart Lung Transplant. (2015) 34:1495–504. 10.1016/j.healun.2015.10.003 26520247

[30] Andrade JG Al-Saloos H Jeewa A Sandor GG Cheung A. Facilitated cardiac recovery in fulminant myocarditis: pediatric use of the Impella LP 5.0 pump. J Heart Lung Transplant. (2010) 29:96–7. 10.1016/j.healun.2009.06.020 19782584

[31] Thuys CA Mullaly RJ Horton SB O'Connor EB Cochrane AD Brizard CP. Centrifugal ventricular assist in children under 6 kg. Eur J Cardiothorac Surg. (1998) 13:130–4. 10.1016/S1010-7940(97)00310-2 9583817

[32] Mohite PN Patil NP Popov AF Bahrami T Simon AR. Oxygenator in short-term LVAD circuit: a rescue in post-LVAD pulmonary complications. Perfusion (2016) 31:608–10. 10.1177/0267659115627691 26791273

[33] Mohite PN Sabashnikov A De Robertis F Popov AF Simon AR. Oxy-RVAD: rescue in pulmonary complications after LVAD implantation. Perfusion (2015) 30:596–9. 10.1177/0267659114566062 25538179

[34] Chou NK Chi NH Wu IW Huang SC Chen YS Yu HY. Extracoporeal membrane oxygenation hybrid with Thoratec ventricular-assist devices as double bridge to heart transplantation. Transplant Proc. (2010) 42:920–2. 10.1016/j.transproceed.2010.02.051 20430204

[35] The Penn State Heart Pump: American Society of Mechanical Engineers (1990). Available online at: https://www.asme.org/wwwasmeorg/media/ResourceFiles/AboutASME/Who%20We%20Are/Engineering%20History/Landmarks/142-Pierce-Donachy-Ventricular-Assist-Device.pdf

[36] Maat AP van Thiel RJ Dalinghaus M Bogers AJ. Connecting the Centrimag Levitronix pump to Berlin Heart Excor cannulae; a new approach to bridge to bridge. J Heart Lung Transplant. (2008) 27:112–5. 10.1016/j.healun.2007.10.010 18187096

[37] Garcia-Guereta L Cabo J de la Oliva P Villar MA Bronte LD Goldman L. Ventricular assist device application with the intermediate use of a membrane oxygenator as a bridge to pediatric heart transplantation. J Heart Lung Transplant. (2009) 28:740–2. 10.1016/j.healun.2009.04.018 19560705

[38] Monge MC Kulat BT Eltayeb O Zingle NR Moss ST Gossett JG. Successful bridge-to-transplant of functionally univentricular patients with a modified continuous-flow ventricular assist device. Artif Organs. (2017) 41:25–31. 10.1111/aor.12881 28093804

[39] Zaccagni HJ Timpa JG O'Meara LC Alten JA. Long-term membrane oxygenator use to support an infant with acute respiratory distress syndrome on biventricular assist device. Interact Cardiovasc Thorac Surg. (2013) 17:196–8. 10.1093/icvts/ivt131 23571680 PMC3686397

[40] De Rita F Hasan A Haynes S Peng E Gandolfo F Ferguson L. Outcome of mechanical cardiac support in children using more than one modality as a bridge to heart transplantation. Eur J Cardiothorac Surg. (2015) 48:917–22; discussion 22. 10.1093/ejcts/ezu544 25605831

[41] Rossano JW Lorts A VanderPluym CJ Jeewa A Guleserian KJ Bleiweis MS. Outcomes of pediatric patients supported with continuous-flow ventricular assist devices: a report from the Pediatric Interagency Registry for Mechanical Circulatory Support (PediMACS). J Heart Lung Transplant. (2016) 35:585–90. 10.1016/j.healun.2016.01.1228 27056612

[42] Nelson-McMillan K Ravekes WJ Thompson WR Brown KM Wolff L Wadia RS. Membrane oxygenator use with biventricular assist device: facilitation of support and lung recovery. World j Pediatric Congenital Heart Surg. (2018) 9:105–9. 10.1177/2150135116668832 27923944

[43] Mansfield RT Lin KY Zaoutis T Mott AR Mohamad Z Luan X. The use of pediatric ventricular assist devices in children's hospitals From 2000 to 2010: morbidity, mortality, and hospital charges. Pediatr Crit Care Med. (2015) 16:522–8. 10.1097/PCC.0000000000000401 25850863

[44] Cove M Maclaren GJ Federspiel W Kellum J. Bench to bedside review: extracorporeal carbon dioxide removal, past present and future. Crit Care. (2012) 16:232. 10.1186/cc11356 23014710 PMC3682237

[45] Vincent JL Annual Update in Intensive Care and Emergency Medicine 2016. New York, NY: Springer international publishing (2016).

[46] Jeffries RG Mussin Y Bulanin DS Lund LW Kocyildirim E Zhumadilov Z. Pre-clinical evaluation of an adult extracorporeal carbon dioxide removal system with active mixing for pediatric respiratory support. Int J Artif Organs. (2014) 37:888–99. 10.5301/ijao.5000372 25588763 PMC5410869

[47] Annich G Adachi I. Anticoagulation for pediatric mechanical circulatory support. Pediatr Crit Care Med. (2013) 14(5 Suppl. 1):S37–42. 10.1097/PCC.0b013e318292dfa7 23735984

[48] Timothy M Maul MPMaPDW. ECMO biocompatibility: surface coatings, anticoagulation, and coagulation monitoring. In: Extracorporeal Membrane Oxygenation - Advances in Therapy. InTech (2016). Available online at: https://www.intechopen.com/books/extracorporeal-membrane-oxygenation-advances-in-therapy/ecmo-biocompatibility-surface-coatings-anticoagulation-and-coagulation-monitoring

[49] Stocker CF Horton SB. Anticoagulation strategies and difficulties in neonatal and paediatric extracorporeal membrane oxygenation (ECMO). Perfusion (2015) 31:95–102. 10.1177/0267659115590626 26060200

[50] Bembea MM Annich G Rycus P Oldenburg G Berkowitz I Pronovost P. Variability in anticoagulation management of patients on extracorporeal membrane oxygenation: an international survey. Pediatr Crit Care Med. (2013) 14:e77–84. 10.1097/PCC.0b013e31827127e4 23287906 PMC3567253

